# Editorial Notes

**Published:** 1928

**Authors:** 


### Editorial Notes.

**Presentation to Dr. D. S. Davies.**

On Wednesday, June 27th, a presentation was made to Dr. D. S. Davies by his medical colleagues in Bristol and the neighbourhood on the occasion of his retirement from the appointment of Medical Officer of Health to the City of Bristol, which he has held for forty-two years.

The presentation took the form of a portable wireless set together with a cheque "for its maintenance," as Dr. Kenneth Wills (President of the Bristol Medico-Chirurgical Society) happily phrased it in offering the gift to Dr. Davies. Dr. Wills expressed the sense of loss that Dr. Davies's retirement imposed on the medical profession. He spoke of the excellent public work he had done, not only as the City Medical Officer of Health, but as a teacher and examiner. Dr. Davies had been President of the Bristol Medico-Chirurgical Society, of the Bath and Bristol Branch of the British Medical Association and of the Society of Medical Officers of Health. He had served as a member of Lord Trevethin's Commission on Venereal Disease in 1922, and had been made an honorary LL.D. of the University of Bristol.

In presenting the wireless set to Dr. Davies on behalf of his colleagues Dr. Wills said he hoped that he would live long to enjoy good health and happiness; and in presenting their small gift he hoped that while "listening in" he might catch from time to time an "atmospheric" of affection and remembrance from his old colleagues and friends.

Dr. J. A. Birrell (Chairman of the Bristol Panel Committee) supported the Chairman in his remarks, and testified to the high estimate his colleagues had formed of Dr. Davies's professional skill, and the help that he was always ready to afford to all who sought his aid.

Professor Hey Groves (President of the Bristol Branch of the British Medical Association) sent apologies for his absence.

Dr. Victoria Tryon (Chairman of the Bristol Division of the British Medical Association) acted as hostess at the tea-table.

Dr. Davies, replying, said that after forty-two years of service it was good to know that he had not forfeited the confidence of his colleagues. He alluded to the great work of Dr. William Budd, and mentioned some of the important outbreaks of epidemic disease he had had to deal with in Bristol, notably the Clifton College outbreak of typhoid fever in 1897 and the small but threatening group of cases of bubonic plague that occurred during the war. He thanked the donors for their kindly gift and the good wishes of which it was the token.

**Royal Society of Medicine.**

ANOTHER member of our Society has had the honour of being elected President of a Section of the Royal Society of Medicine, and we offer our hearty congratulations to Mr. C. H. Walker, who will preside over the Section of Ophthalmology during the coming year. Three other members of the Society have already been Presidents of Sections of the Royal Society of Medicine, namely Dr. Patrick Watson-Williams (Laryngology and Otology, 1910–11), Mr. W. R. Ackland (Odontology, 1922–23) and Dr. A. L. Flemming (Anæsthetics, 1922–23 and 1923–24).

**Students' Number.**

THIS issue of the Journal might almost be described as a students' number, because five of the original articles have been contributed by our students. One (that by Miss Vine) was an essay which won a British Medical Association Prize. Critchley's paper on the ocular accompaniments of encephalitis lethargica gained for the writer the Barrett Roué Prize at the Bristol General Hospital, and the essays by Pyke and Fisher were successfully offered for the Martyn Memorial Scholarships. If these papers had merely been embodiments of reading they would not have been considered worthy of publication, but as they are in each instance examples of first-hand observations they merit record. Our readers may recall that Sir James Paget discovered the trichina spiralis when he was a first-year student in the dissecting-room of St. Bartholomew's Hospital.

**Colston Research Society.**

THE Annual Dinner of the Colston Research Society was held at the Victoria Rooms, Clifton, on Friday, May 18th, under the Presidency of Mr. Reginald Earle, and was attended by about 100 guests. The Society was to have entertained Sir Alfred Mond, whose visit was looked forward to with much interest, but at the last moment he found himself unable to get away from London owing to important business negotiations demanding his immediate attention, and in his place be sent Major A. E. Freeth, F.R.S., Scientific Adviser to Imperial Chemical Industries. Major Freeth, responding to the toast of "Our Guest," proposed by Dr. Stanley Badock, Chairman of Council of the University, delivered the speech which would have been given by Sir Alfred, and what he had to say was listened to with close attention by those present.

The collection was announced at £812 3s. 0d., £200 of which has been earmarked by the donors, as in the previous year, for the maintenance of the Colston Research Fellowship in connection with the University Centre of Cardiac Research at the Bristol General Hospital.

Sir Alfred Mond's speech supported very warmly the aims and objects of the Society, and pointed out that Bristol now has at her disposal a proved organisation which, properly supported, would make her a leader in Research. At the present moment the number of business firms subscribing annually is under 60, out of a total of about 300 subscribers, and one cannot but feel that the efforts of the Society are such as should receive more serious consideration and a very largely increased measure of financial support from the commercial community of Bristol. This point was urged strongly by the President in response to the toast of his health, proposed by the Lord Mayor, and also by the Hon. Secretary of the Society, who was present in his capacity as Sheriff of Bristol.

